# Supplementary material for: Functionally constrained human proteins are less prone to mutational instability from single amino acid substitutions
Source: Nat Commun. 2025 Mar 13;16:2492. doi: 10.1038/s41467-025-57757-y (PMC11906876; doi:10.1038/s41467-025-57757-y)
Supplement: Supplementary file 1 — Supplementary Information [file 41467_2025_57757_MOESM1_ESM.pdf]

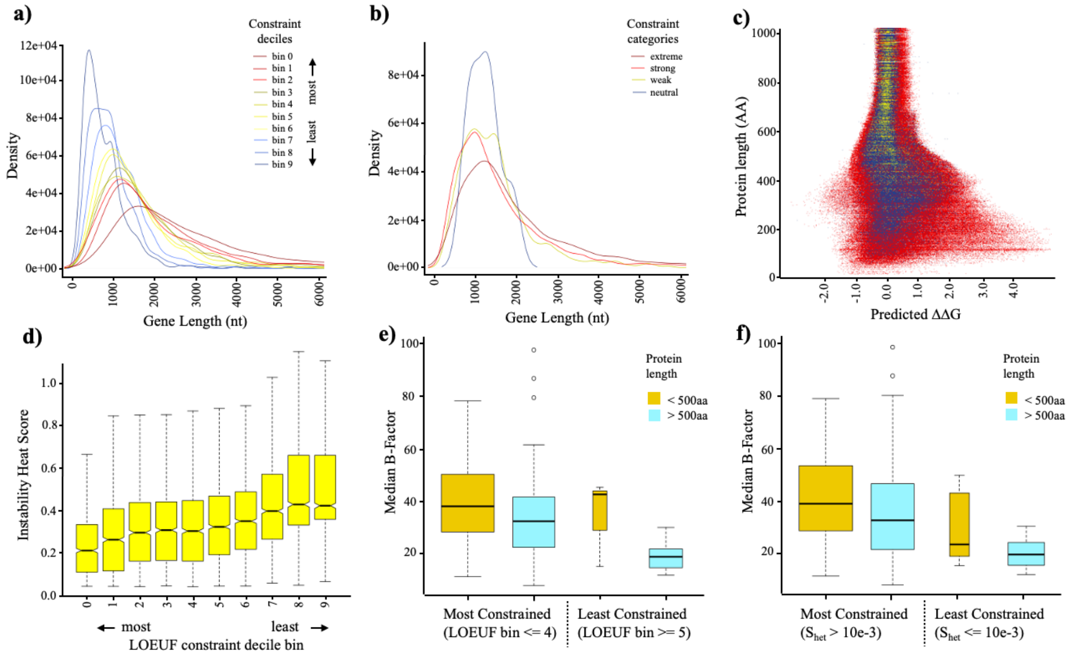

Supplementary Figure 1 – Relationship between gene/protein length and predicted ΔΔG. Density plots of gene length versus functional constraint categories for **a)** GnomAD LOEUF constraint deciles, and **b)** S_{het} constraint categories. **c)** Predicted ΔΔG values for all possible amino acid substitutions (red), GnomAD observed substitutions (blue) and observed substitutions in most constrained proteins (GnomAD LOEUF bin 0; yellow) by protein length. For all following boxplots, the central boxplot bar is the median value of each respective category, the bounds of each box are the interquartile range (IQR), whiskers extend 1.5*IQR from each box and the central notches (where present) represent an approximation of the 95% confidence interval of the median box value. **d)** Distributions of Instability Heat Score for all human proteins, grouped by LOEUF functional constraint decile bins. In this boxplot, Instability heat scores are calculated with variable ΔΔG thresholds as a function of protein length. Thresholds applied were as follows (format: length_range;lowerΔΔGthresh; upperΔΔGthresh): 1-249,-0.25,0.50;250-299,-0.25,1.00;300-349,-0.25,1.25;350-449,-0.5,1.00;450-499,-0.5,0.75;500-549,-0.75,0.5;550-599,-0.75,0.25;600-699,-0.50,0.25;700-1000,-0.25,0.25). Within each LOEUF constraint decile bin, the sample sizes were as follows: bin 0 n=1111, bin 1 n=1421, bin 2 n=1469, bin 3 n=1523, bin 4 n=1540, bin 5 n=1578, bin 6 n=1657, bin 7 n=1683, bin 8 n=1623, bin 9 n=1392. **e)** Boxplot of relative distributions of median B-factor for experimentally solved crystal structures grouped by functional constraint with LOEUF deciles (Most Constrained n=51, Least Constrained n=33), segregated by protein lengths. **f)** Boxplot of relative distributions of median B-factor by S_{het} categories (Most Constrained n=65; Least Constrained n=26), segregated by protein lengths. Source data are provided as a Source Data file.

Supplementary Table 1 – Details of experimentally solved protein structures.

Protein	PDB_ID	AA_range	PartialStructure	Reference
ACTN3	1WKU	26-273	Y	1
ACTN4	2R0O	46-272	Y	2
ACVRL1	3MY0	195-494	Y	3
AGR3	3PH9	31-165	Y	4
AKR7A2	2BP1	38-360	Y	5
AKT3	2X18	4-118		6
AMY2A	1CPU	2-496		7
AP4M1	3L81	185-692	Y	8
AR	1E3G	669-918	Y	9
ATM	5NP0	1-3056		10
BMPR2	2HLQ	32-131	Y	11
BTK	1K2P	397-654	Y	12
CD1C	5C9J	24-203	Y	13
CDKL2	4AAA	1-308	Y	14
CPB1	1KWM	4-309		15
CRYGB	2JDG	1-172		16
CRYZ	1YB5	6-329		17
CTSV	1FH0	114-334	Y	18
CUL4B	2B5L	1-999		19
DDX3X	2I4I	167-580	Y	20
EPHB2	3ZFM	615-894	Y	21
EZH2	5HYN	20-736		22
FIT5	3ZGQ	1-482		23
FOXK2	2C6Y	256-353	Y	24
GALK2	2A2C	1-456		25
GBP1	1F5N	7-583		26
GCK	1V4S	14-460		27
GH1	1A22	27-217		28
GIPR	2QKH	1-122		29
GJB2	5ER7	33-197	Y	30
HNF1B	2H8R	90-310	Y	31
ICAM3	1T0P	127-300	Y	32
IL18RAP	2M1W	75-235	Y	33
KIF5A	4UXT	1-426		34
LCK	3KXZ	225-509	Y	35
LDLR	1N7D	22-720		36
LRP8	2P4E	61-682	Y	37
MCOLN1	5TJA	82-293	Y	38
MEN1	3U84	1-608		39
MIB1	4XI6	7-400		40
MMP8	1JAP	86-242	Y	41

MSH3	3THX	15-999		42
MYH7	4PA0	2-999		43
NF2	1H4R	20-313		44
NFKBIA	1IKN	19-357		45
NRAS	5UHV	1-166		46
NSUN2	4FZV	26-384		47
NUDT14	3Q91	28-222		48
OGFOD1	4NHX	1-542		49
PADI4	2DEW	3-663		50
PAH	1DMW	118-424	Y	51
PARK7	1PDV	2-188		52
PLA3G1B	3ELO	1-270		53
POLG	3IKM	70-999	Y	54
PPP3CB	4OR9	16-493		55
PTEN	5BZZ	14-351		56
PTPN11	2SHP	2-525		57
RAD21	4PJU	84-999		58
RARB	1XAP	175-409	Y	59
RBPJ	2F8X	1-432		60
REG3A	4MTH	37-175	Y	61
RPAP3	6GXZ	281-445	Y	62
RPS6KA3	4D9T	418-713	Y	63
SEC14L3	4UYB	1-400		64
SERPINC1	1ANT	18-430		65
SGSH	4MHX	22-504		66
SLC2A1	4PYP	16-455		67
SPAST	3VFD	324-613	Y	68
STAT1	1YVL	2-683		69
SULT1C2	3BFX	12-296		70
TBL1XR1	4LG9	152-514	Y	71
TFRC	3KAS	121-760	Y	72
TNFAIP3	2VFJ	5-355		73
TNNI3K	4YFI	441-729	Y	74
TNPO1	1QBK	19-890		75
TOP1	1A36	1-765		76
TP53	1UOL	96-290	Y	77
TRAF3	1FLK	300-504	Y	78
TYK2	4OLI	579-988	Y	79
TYMP	1UOU	33-480		80
UBA2	1Y8Q	10-550		81
VCP	3HU3	17-469		82
ZMYND11	4NS5	156-364	Y	83

Supplementary References

1. Franzot, G., Sjöblom, B., Gautel, M., & Carugo, K. D. (2005). The crystal structure of the actin binding domain from α-actinin in its closed conformation: structural insight into phospholipid regulation of α-actinin. *Journal of molecular biology*, *348*(1), 151-165.

2. Lee, S. H., Weins, A., Hayes, D. B., Pollak, M. R., & Dominguez, R. (2008). Crystal structure of the actin-binding domain of α-actinin-4 Lys255Glu mutant implicated in focal segmental glomerulosclerosis. *Journal of molecular biology*, *376*(2), 317-324.

3. Kerr, G., Sheldon, H., Chaikuad, A., Alfano, I., von Delft, F., Bullock, A. N., & Harris, A. L. (2015). A small molecule targeting ALK1 prevents Notch cooperativity and inhibits functional angiogenesis. *Angiogenesis*, *18*, 209-217.

4. Nguyen, V. D., Biterova, E., Salin, M., Wierenga, R. K., & Ruddock, L. W. (2018). Crystal structure of human anterior gradient protein 3. *Acta Crystallographica Section F: Structural Biology Communications*, *74*(7), 425-430.

5. https://dx.doi.org/10.2210/pdb2bp1/pdb

6. https://dx.doi.org/10.2210/pdb2x18/pdb

7. Brayer, G. D., Sidhu, G., Maurus, R., Rydberg, E. H., Braun, C., Wang, Y., ... & Withers, S. G. (2000). Subsite mapping of the human pancreatic α-amylase active site through structural, kinetic, and mutagenesis techniques. *Biochemistry*, *39*(16), 4778-4791.

8. Burgos, P. V., Mardones, G. A., Rojas, A. L., Luis, L. P., Prabhu, Y., Hurley, J. H., & Bonifacino, J. S. (2010). Sorting of the Alzheimer's disease amyloid precursor protein mediated by the AP-4 complex. *Developmental cell*, *18*(3), 425-436.

9. Matias, P. M., Donner, P., Coelho, R., Thomaz, M., Peixoto, C., Macedo, S., ... & Carrondo, M. A. (2000). Structural evidence for ligand specificity in the binding domain of the human androgen receptor: implications for pathogenic gene mutations. *Journal of Biological Chemistry*, *275*(34), 26164-26171.

10. Baretić, D., Pollard, H. K., Fisher, D. I., Johnson, C. M., Santhanam, B., Truman, C. M., ... & Williams, R. L. (2017). Structures of closed and open conformations of dimeric human ATM. *Science advances*, *3*(5), e1700933.

11. Mace, P. D., Cutfield, J. F., & Cutfield, S. M. (2006). High resolution structures of the bone morphogenetic protein type II receptor in two crystal forms: implications for ligand binding. *Biochem. Biophys. Res. Commun*., 351(4), 831-8.

12. Mao, C., Zhou, M., & Uckun, F. M. (2001). Crystal structure of Bruton's tyrosine kinase domain suggests a novel pathway for activation and provides insights into the molecular basis of X-linked agammaglobulinemia. *J. Biol. Chem*., 276(44), 41435-43.

13. Mansour, S., Tocheva, A. S., Cave-Ayland, C., Machelett, M. M., Sander, B., Lissin, N. M., et al. (2016). Cholesteryl esters stabilize human CD1c conformations for recognition by self-reactive T cells. *Proc. Natl. Acad. Sci. U.S.A.,* 113(9), E1266-75.

14. Canning, P., Park, K., Gonçalves, J., Li, C., Howard, C. J., Sharpe, T. D., et al. (2018). CDKL Family Kinases Have Evolved Distinct Structural Features and Ciliary Function. *Cell Rep*, 22(4), 885-894.

15. Barbosa Pereira, P. J., Segura-Martín, S., Oliva, B., Ferrer-Orta, C., Avilés, F. X., Coll, M., et al. (2002). Human procarboxypeptidase B: three-dimensional structure and implications for thrombin-activatable fibrinolysis inhibitor (TAFI). *J. Mol. Biol*., 321(3), 537-47.

16. Ebersbach, H., Fiedler, E., Scheuermann, T., Fiedler, M., Stubbs, M. T., Reimann, C., et al. (2007). Affilin-novel binding molecules based on human gamma-B-crystallin, an all beta-sheet protein. *J. Mol. Biol.,* 372(1), 172-85.

17. https://dx.doi.org/10.2210/pdb1yb5/pdb

18. Somoza, J. R., Zhan, H., Bowman, K. K., Yu, L., Mortara, K. D., Palmer, J. T., et al. (2000). Crystal structure of human cathepsin V. Biochemistry, 39(41), 12543-51.

19. Li, T., Chen, X., Garbutt, K. C., Zhou, P., & Zheng, N. (2006). Structure of DDB1 in complex with a paramyxovirus V protein: viral hijack of a propeller cluster in ubiquitin ligase. Cell, 124(1), 105-17.

20. Högbom, M., Collins, R., van den Berg, S., Jenvert, R. M., Karlberg, T., Kotenyova, T., et al. (2007). Crystal structure of conserved domains 1 and 2 of the human DEAD-box helicase DDX3X in complex with the mononucleotide AMP. J. Mol. Biol., 372(1), 150-9.

21. Overman, R. C., Debreczeni, J. E., Truman, C. M., McAlister, M. S., & Attwood, T. K. (2014). Completing the structural family portrait of the human EphB tyrosine kinase domains. Protein Sci., 23(5), 627-38.

22. Justin, N., Zhang, Y., Tarricone, C., Martin, S. R., Chen, S., Underwood, E., et al. (2016). Structural basis of oncogenic histone H3K27M inhibition of human polycomb repressive complex 2. Nat Commun, 7, 11316.

23. Katibah, G. E., Lee, H. J., Huizar, J. P., Vogan, J. M., Alber, T., & Collins, K. (2013). tRNA binding, structure, and localization of the human interferon-induced protein IFIT5. Mol. Cell, 49(4), 743-50.

24. Tsai, K. L., Huang, C. Y., Chang, C. H., Sun, Y. J., Chuang, W. J., & Hsiao, C. D. (2006). Crystal structure of the human FOXK1a-DNA complex and its implications on the diverse binding specificity of winged helix/forkhead proteins. J. Biol. Chem., 281(25), 17400-17409.

25. Thoden, J. B., & Holden, H. M. (2005). The molecular architecture of human N-acetylgalactosamine kinase. J. Biol. Chem., 280(38), 32784-91.

26. Prakash, B., Renault, L., Praefcke, G. J., Herrmann, C., & Wittinghofer, A. (2000). Triphosphate structure of guanylate-binding protein 1 and implications for nucleotide binding and GTPase mechanism. EMBO J., 19(17), 4555-64.

27. Kamata, K., Mitsuya, M., Nishimura, T., Eiki, J., & Nagata, Y. (2004). Structural basis for allosteric regulation of the monomeric allosteric enzyme human glucokinase. Structure, 12(3), 429-38.

28. Clackson, T., Ultsch, M. H., Wells, J. A., & de Vos, A. M. (1998). Structural and functional analysis of the 1:1 growth hormone:receptor complex reveals the molecular basis for receptor affinity. J. Mol. Biol., 277(5), 1111-28.

29. Parthier, C., Kleinschmidt, M., Neumann, P., Rudolph, R., Manhart, S., Schlenzig, D., et al. (2007). Crystal structure of the incretin-bound extracellular domain of a G protein-coupled receptor. Proc. Natl. Acad. Sci. U.S.A., 104(35), 13942-7.

30. Bennett, B. C., Purdy, M. D., Baker, K. A., Acharya, C., McIntire, W. E., Stevens, R. C., et al. (2016). An electrostatic mechanism for Ca(2+)-mediated regulation of gap junction channels. Nat Commun, 7, 8770.

31. Lu, P., Rha, G. B., & Chi, Y. I. (2007). Structural basis of disease-causing mutations in hepatocyte nuclear factor 1beta. Biochemistry, 46(43), 12071-80.

32. Song, G., Yang, Y., Liu, J. H., Casasnovas, J. M., Shimaoka, M., Springer, T. A., et al. (2005). An atomic resolution view of ICAM recognition in a complex between the binding domains of ICAM-3 and integrin alphaLbeta2. Proc. Natl. Acad. Sci. U.S.A., 102(9), 3366-71.

33. Enokizono, Y., Kumeta, H., Funami, K., Horiuchi, M., Sarmiento, J., Yamashita, K., et al. (2013). Structures and interface mapping of the TIR domain-containing adaptor molecules involved in interferon signaling. Proc. Natl. Acad. Sci. U.S.A., 110(49), 19908-13.

34. Atherton, J., Farabella, I., Yu, I. M., Rosenfeld, S. S., Houdusse, A., Topf, M., et al. (2014). Conserved mechanisms of microtubule-stimulated ADP release, ATP binding, and force generation in transport kinesins. Elife, 3, e03680.

35. Moy, F. J., Lee, A., Gavrin, L. K., Xu, Z. B., Sievers, A., Kieras, E., et al. (2010). Novel synthesis and structural characterization of a high-affinity paramagnetic kinase probe for the identification of non-ATP site binders by nuclear magnetic resonance. J. Med. Chem., 53(3), 1238-49.

36. Rudenko, G., Henry, L., Henderson, K., Ichtchenko, K., Brown, M. S., Goldstein, J. L., et al. (2002). Structure of the LDL receptor extracellular domain at endosomal pH. Science, 298(5602), 2353-8.

37. Cunningham, D., Danley, D. E., Geoghegan, K. F., Griffor, M. C., Hawkins, J. L., Subashi, T. A., et al. (2007). Structural and biophysical studies of PCSK9 and its mutants linked to familial hypercholesterolemia. Nat. Struct. Mol. Biol., 14(5), 413-9.

38. Li, M., Zhang, W. K., Benvin, N. M., Zhou, X., Su, D., Li, H., et al. (2017). Structural basis of dual Ca2+/pH regulation of the endolysosomal TRPML1 channel. Nat. Struct. Mol. Biol., 24(3), 205-213.

39. Huang, J., Gurung, B., Wan, B., Matkar, S., Veniaminova, N. A., Wan, K., et al. (2012). The same pocket in menin binds both MLL and JUND but has opposite effects on transcription. Nature, 482(7386), 542-6.

40. McMillan, B. J., Schnute, B., Ohlenhard, N., Zimmerman, B., Miles, L., Beglova, N., et al. (2015). A tail of two sites: a bipartite mechanism for recognition of notch ligands by mind bomb E3 ligases. Mol. Cell, 57(5), 912-924.

41. Bode, W., Reinemer, P., Huber, R., Kleine, T., Schnierer, S., & Tschesche, H. (1994). The X-ray crystal structure of the catalytic domain of human neutrophil collagenase inhibited by a substrate analogue reveals the essentials for catalysis and specificity. EMBO J., 13(6), 1263-9.

42. Gupta, S., Gellert, M., & Yang, W. (2011). Mechanism of mismatch recognition revealed by human MutSβ bound to unpaired DNA loops. Nat. Struct. Mol. Biol., 19(1), 72-8.

43. Winkelmann, D. A., Forgacs, E., Miller, M. T., & Stock, A. M. (2015). Structural basis for drug-induced allosteric changes to human β-cardiac myosin motor activity. Nat Commun, 6, 7974.

44. Kang, B. S., Cooper, D. R., Devedjiev, Y., Derewenda, U., & Derewenda, Z. S. (2002). The structure of the FERM domain of merlin, the neurofibromatosis type 2 gene product. Acta Crystallogr. D Biol. Crystallogr., 58(Pt 3), 381-91.

45. Huxford, T., Huang, D. B., Malek, S., & Ghosh, G. (1998). The crystal structure of the IkappaBalpha/NF-kappaB complex reveals mechanisms of NF-kappaB inactivation. Cell, 95(6), 759-70.

46. Johnson, C. W., Reid, D., Parker, J. A., Salter, S., Knihtila, R., Kuzmic, P., et al. (2017). The small GTPases K-Ras, N-Ras, and H-Ras have distinct biochemical properties determined by allosteric effects. J. Biol. Chem., 292(31), 12981-12993.

47. Yakubovskaya, E., Guja, K. E., Mejia, E., Castano, S., Hambardjieva, E., Choi, W. S., et al. (2012). Structure of the essential MTERF4:NSUN4 protein complex reveals how an MTERF protein collaborates to facilitate rRNA modification. Structure 20:1940-7.

48. https://dx.doi.org/10.2210/pdb3q91/pdb

49. Horita, S., Scotti, J. S., Thinnes, C., Mottaghi-Taromsari, Y. S., Thalhammer, A., Ge, W., et al. (2015). Structure of the ribosomal oxygenase OGFOD1 provides insights into the regio- and stereoselectivity of prolyl hydroxylases. Structure, 23(4), 639-52.

50. Arita, K., Shimizu, T., Hashimoto, H., Hidaka, Y., Yamada, M., & Sato, M. (2006). Structural basis for histone N-terminal recognition by human peptidylarginine deiminase 4. Proc. Natl. Acad. Sci. U.S.A., 103(14), 5291-6.

51. Erlandsen, H., Bjørgo, E., Flatmark, T., & Stevens, R. C. (2000). Crystal structure and site-specific mutagenesis of pterin-bound human phenylalanine hydroxylase. Biochemistry, 39(9), 2208-17.

52. Tao, X., & Tong, L. (2003). Crystal structure of human DJ-1, a protein associated with early onset Parkinson's disease. J. Biol. Chem., 278(33), 31372-9.

53. Xu, W., Yi, L., Feng, Y., Chen, L., & Liu, J. (2009). Structural insight into the activation mechanism of human pancreatic prophospholipase A2. J. Biol. Chem., 284(24), 16659-16666.

54. Lee, Y. S., Kennedy, W. D., & Yin, Y. W. (2009). Structural insight into processive human mitochondrial DNA synthesis and disease-related polymerase mutations. Cell, 139(2), 312-24.

55. https://dx.doi.org/10.2210/pdb4or9/pdb

56. Lee, C. U., Hahne, G., Hanske, J., Bange, T., Bier, D., Rademacher, C., et al. (2015). Redox Modulation of PTEN Phosphatase Activity by Hydrogen Peroxide and Bisperoxidovanadium Complexes. Angew. Chem. Int. Ed. Engl., 54(46), 13796-800.

57. Hof, P., Pluskey, S., Dhe-Paganon, S., Eck, M. J., & Shoelson, S. E. (1998). Crystal structure of the tyrosine phosphatase SHP-2. Cell, 92(4), 441-50.

58. Hara, K., Zheng, G., Qu, Q., Liu, H., Ouyang, Z., Chen, Z., et al. (2014). Structure of cohesin subcomplex pinpoints direct shugoshin-Wapl antagonism in centromeric cohesion. Nat. Struct. Mol. Biol., 21(10), 864-70.

59. Germain, P., Kammerer, S., Pérez, E., Peluso-Iltis, C., Tortolani, D., Zusi, F. C., et al. (2004). Rational design of RAR-selective ligands revealed by RARbeta crystal stucture. EMBO Rep., 5(9), 877-82.

60. Nam, Y., Sliz, P., Song, L., Aster, J. C., & Blacklow, S. C. (2006). Structural basis for cooperativity in recruitment of MAML coactivators to Notch transcription complexes. Cell, 124(5), 973-83.

61. Mukherjee, S., Zheng, H., Derebe, M. G., Callenberg, K. M., Partch, C. L., Rollins, D., et al. (2014). Antibacterial membrane attack by a pore-forming intestinal C-type lectin. Nature, 505(7481), 103-7.

62. Henri, J., Chagot, M. E., Bourguet, M., Abel, Y., Terral, G., Maurizy, C., et al. (2018). Deep Structural Analysis of RPAP3 and PIH1D1, Two Components of the HSP90 Co-chaperone R2TP Complex. Structure, 26(9), 1196-1209.e8.

63. Serafimova, I. M., Pufall, M. A., Krishnan, S., Duda, K., Cohen, M. S., Maglathlin, R. L., et al. (2012). Reversible targeting of noncatalytic cysteines with chemically tuned electrophiles. Nat. Chem. Biol., 8(5), 471-6.

64. https://dx.doi.org/10.2210/pdb4uyb/pdb

65. Carrell, R. W., Stein, P. E., Fermi, G., & Wardell, M. R. (1994). Biological implications of a 3 A structure of dimeric antithrombin. Structure, 2(4), 257-70.

66. Sidhu, N. S., Schreiber, K., Pröpper, K., Becker, S., Usón, I., Sheldrick, G. M., et al. (2014). Structure of sulfamidase provides insight into the molecular pathology of mucopolysaccharidosis IIIA. Acta Crystallogr. D Biol. Crystallogr., 70(Pt 5), 1321-35.

67. Deng, D., Xu, C., Sun, P., Wu, J., Yan, C., Hu, M., et al. (2014). Crystal structure of the human glucose transporter GLUT1. Nature, 510(7503), 121-5.

68. Taylor, J. L., White, S. R., Lauring, B., & Kull, F. J. (2012). Crystal structure of the human spastin AAA domain. J. Struct. Biol., 179(2), 133-7.

69. Mao, X., Ren, Z., Parker, G. N., Sondermann, H., Pastorello, M. A., Wang, W., et al. (2005). Structural bases of unphosphorylated STAT1 association and receptor binding. Mol. Cell, 17(6), 761-71.

70. Dombrovski, L., Dong, A., Bochkarev, A., & Plotnikov, A. N. (2006). Crystal structures of human sulfotransferases SULT1B1 and SULT1C1 complexed with the cofactor product adenosine-3'-5'-diphosphate (PAP). *Proteins*, *64*(4), 1091-1094.

71. https://dx.doi.org/10.2210/pdb4lg9/pdb

72. Abraham, J., Corbett, K. D., Farzan, M., Choe, H., & Harrison, S. C. (2010). Structural basis for receptor recognition by New World hemorrhagic fever arenaviruses. Nat. Struct. Mol. Biol., 17(4), 438-44.

73. Komander, D., & Barford, D. (2008). Structure of the A20 OTU domain and mechanistic insights into deubiquitination. Biochem. J., 409(1), 77-85.

74. Lawhorn, B. G., Philp, J., Zhao, Y., Louer, C., Hammond, M., Cheung, M., et al. (2015). Identification of Purines and 7-Deazapurines as Potent and Selective Type I Inhibitors of Troponin I-Interacting Kinase (TNNI3K). J. Med. Chem., 58(18), 7431-48.

75. Chook, Y. M., & Blobel, G. (1999). Structure of the nuclear transport complex karyopherin-beta2-Ran x GppNHp. Nature, 399(6733), 230-7.

76. Stewart, L., Redinbo, M. R., Qiu, X., Hol, W. G., & Champoux, J. J. (1998). A model for the mechanism of human topoisomerase I. Science, 279(5356), 1534-41.

77. Joerger, A. C., Allen, M. D., & Fersht, A. R. (2004). Crystal structure of a superstable mutant of human p53 core domain. Insights into the mechanism of rescuing oncogenic mutations. J. Biol. Chem., 279(2), 1291-6.

78. Ni, C. Z., Welsh, K., Leo, E., Chiou, C. K., Wu, H., Reed, J. C., et al. (2000). Molecular basis for CD40 signaling mediated by TRAF3. Proc. Natl. Acad. Sci. U.S.A., 97(19), 10395-9.

79. Lupardus, P. J., Ultsch, M., Wallweber, H., Bir Kohli, P., Johnson, A. R., & Eigenbrot, C. (2014). Structure of the pseudokinase-kinase domains from protein kinase TYK2 reveals a mechanism for Janus kinase (JAK) autoinhibition. Proc. Natl. Acad. Sci. U.S.A., 111(22), 8025-30.

80. Norman, R. A., Barry, S. T., Bate, M., Breed, J., Colls, J. G., Ernill, R. J., et al. (2004). Crystal structure of human thymidine phosphorylase in complex with a small molecule inhibitor. Structure, 12(1), 75-84.

81. Lois, L. M., & Lima, C. D. (2005). Structures of the SUMO E1 provide mechanistic insights into SUMO activation and E2 recruitment to E1. EMBO J., 24(3), 439-51.

82. Tang, W. K., Li, D., Li, C. C., Esser, L., Dai, R., Guo, L., et al. (2010). A novel ATP-dependent conformation in p97 N-D1 fragment revealed by crystal structures of disease-related mutants. EMBO J., 29(13), 2217-29.

83. Wang, J., Qin, S., Li, F., Li, S., Zhang, W., Peng, J., et al. (2014). Crystal structure of human BS69 Bromo-ZnF-PWWP reveals its role in H3K36me3 nucleosome binding. Cell Res., 24(7), 890-3.
