# Supplementary material for: Functionally constrained human proteins are less prone to mutational instability from single amino acid substitutions
Source: Nat Commun. 2025 Mar 13;16:2492. doi: 10.1038/s41467-025-57757-y (PMC11906876; doi:10.1038/s41467-025-57757-y)
Supplement: Supplementary file 2 — Reporting Summary [file 41467_2025_57757_MOESM2_ESM.pdf]

nature portfolio

Corresponding author(s): TD Andrews

Last updated by author(s): 25/01/25

Reporting Summary

Nature Portfolio wishes to improve the reproducibility of the work that we publish. This form provides structure for consistency and transparency in reporting. For further information on Nature Portfolio policies, see our Editorial Policies and the Editorial Policy Checklist.

Statistics

For all statistical analyses, confirm that the following items are present in the figure legend, table legend, main text, or Methods section.

n/a	Confirmed	
[ ]	[x]	The exact sample size (*n*) for each experimental group/condition, given as a discrete number and unit of measurement
[x]	[ ]	A statement on whether measurements were taken from distinct samples or whether the same sample was measured repeatedly
[x]	[ ]	The statistical test(s) used AND whether they are one- or two-sided<br>*Only common tests should be described solely by name; describe more complex techniques in the Methods section.*
[x]	[ ]	A description of all covariates tested
[x]	[ ]	A description of any assumptions or corrections, such as tests of normality and adjustment for multiple comparisons
[ ]	[x]	A full description of the statistical parameters including central tendency (e.g. means) or other basic estimates (e.g. regression coefficient) AND variation (e.g. standard deviation) or associated estimates of uncertainty (e.g. confidence intervals)
[x]	[ ]	For null hypothesis testing, the test statistic (e.g. *F*, *t*, *r*) with confidence intervals, effect sizes, degrees of freedom and *P* value noted<br>*Give P values as exact values whenever suitable.*
[x]	[ ]	For Bayesian analysis, information on the choice of priors and Markov chain Monte Carlo settings
[x]	[ ]	For hierarchical and complex designs, identification of the appropriate level for tests and full reporting of outcomes
[x]	[ ]	Estimates of effect sizes (e.g. Cohen's *d*, Pearson's *r*), indicating how they were calculated

Our web collection on statistics for biologists contains articles on many of the points above.

Software and code

Policy information about availability of computer code

Data collection	Human population missense variation was obtained from GnomAD v4.1 (ref. 12,28). Canonical transcripts for human genes were obtained from EnsEMBL47 release GRCh38 p14. Variant calls from disease-free individual genomes were obtained from the 1000 Genomes, Phase 3 release (ref 20). Annotated pathogenic and benign missense variants were obtained from the ClinVar database(ref. 19) release 230930. Predictions of the stability effects of all possible single amino acid substitutions were made using structural models available from the AlphaFold2 Protein Structure Database (ref. 17). The results of MAESTRO predictions (ref. 16) made for all human proteins(ref. 15) are available at www.stabilitysort.org/download/. 11198 de novo, missense mutations identified among ASD probands were obtained from the original work describing these (see Supplementary Table 20 in Fu et al ref. 31). Observed and expected pLoF variant counts and LOEUF scores were obtained from GnomAD (ref. 12). AlphaMissense median pathogenicity scores were obtained from Cheng et a ref. l21). Shet scores were obtained from the supplementary data of Zeng et al (ref. 14).

Data analysis	LOEUF Calculation<br>To calculate the expected number of missense DoF variants and the LOEUF score for a given gene, a minor extension was made to existing methodology (ref. 12,25). Briefly, for each nucleotide site in a transcript, potential missense variants were appraised according to the structural stability effects of the amino acid substitution. For a substitution with predicted stability effects greater than defined thresholds (in most cases -0.5 < DDG < 0.5, see Results), the substitution was counted as missense DoF variation. These expected missense DoF variants were then used in place of expected LoF variants for calculating LOUEF values for each gene. Likewise, for observed variants, the predicted stability effects of each observed missense variant were appraised and counted if outside of the threshold values being applied. A python implementation of this method using missense DoF variation is available from https://gitlab.com/tdaadt/missensedof.

For manuscripts utilizing custom algorithms or software that are central to the research but not yet described in published literature, software must be made available to editors and reviewers. We strongly encourage code deposition in a community repository (e.g. GitHub). See the Nature Portfolio guidelines for submitting code & software for further information.

Data

Policy information about availability of data

All manuscripts must include a data availability statement. This statement should provide the following information, where applicable:

- Accession codes, unique identifiers, or web links for publicly available datasets
- A description of any restrictions on data availability
- For clinical datasets or third party data, please ensure that the statement adheres to our policy

Data Availability
All datasets used in this study are available in the public domain, including the comprehensive set of stability change predictions for all possible single amino acid substitutions in human proteins (www.stabilitysort.org/download). Supplementary Data 1 (doi.org/10.5281/zenodo.14724237) provides DoS metrics for each human gene with a 1-to-1 mapping between an EnsEMBL transcript (GRCh38 p14; www.ensembl.org) and UniProt protein identifier (www.uniprot.org) with a corresponding AlphaFold2 predicted structure (www.alphafold.ebi.ac.uk). Source data are provided with this paper.

Research involving human participants, their data, or biological material

Policy information about studies with human participants or human data. See also policy information about sex, gender (identity/presentation), and sexual orientation and race, ethnicity and racism.

Reporting on sex and gender	Not applicable to this study
Reporting on race, ethnicity, or other socially relevant groupings	Not applicable to this study
Population characteristics	Not applicable to this study
Recruitment	Only public datasets have been analysed in this study
Ethics oversight	Only public datasets have been analysed in this study

Note that full information on the approval of the study protocol must also be provided in the manuscript.

Field-specific reporting

Please select the one below that is the best fit for your research. If you are not sure, read the appropriate sections before making your selection.

[x] Life sciences [ ] Behavioural & social sciences [ ] Ecological, evolutionary & environmental sciences

For a reference copy of the document with all sections, see nature.com/documents/nr-reporting-summary-flat.pdf

Life sciences study design

All studies must disclose on these points even when the disclosure is negative.

Sample size	Sample size was dictated by available public datasets, such as the GnomAD 4.1 and present ClinVar data. We note that there continues to be debate about whether these are sufficiently powered for, say, correct identification of functionally constrained genes (eg. see ref. 14). This is a central aspect and motivator for this work. The final conclusion of the Abstract posits that DoS variation replicates the patterns of functional constraint from previous data and that these will provide better powered datasets for estimating functional constraint.
Data exclusions	No data was excluded or removed from the full datasets available to this study
Replication	This study used the largest available population dataset of human genetic variation, and presently this is not yet replicated as a public resource.
Randomization	Population-scale characteristics of genetic variation in distinct genes only was appraised and this was not further stratified into groups or cohorts

Blinding	Cohorts or groups were not used to stratify data and, hence, did not require blinding

Reporting for specific materials, systems and methods

We require information from authors about some types of materials, experimental systems and methods used in many studies. Here, indicate whether each material, system or method listed is relevant to your study. If you are not sure if a list item applies to your research, read the appropriate section before selecting a response.

Materials & experimental systems

n/a	Involved in the study
☒	☐ Antibodies
☒	☐ Eukaryotic cell lines
☒	☐ Palaeontology and archaeology
☒	☐ Animals and other organisms
☒	☐ Clinical data
☒	☐ Dual use research of concern
☒	☐ Plants

Methods

n/a	Involved in the study
☒	☐ ChIP-seq
☒	☐ Flow cytometry
☒	☐ MRI-based neuroimaging

Plants

Seed stocks	Plants not the subject of this study
Novel plant genotypes	Plants not the subject of this study
Authentication	Plants not the subject of this study
